# Multiple input algorithm-guided Deep Brain stimulation-programming for Parkinson’s disease patients

**DOI:** 10.1038/s41531-022-00396-7

**Published:** 2022-10-29

**Authors:** Eileen Gülke, León Juárez Paz, Heleen Scholtes, Christian Gerloff, Andrea A. Kühn, Monika Pötter-Nerger

**Affiliations:** 1 Department of Neurology, University Medical Center Hamburg-Eppendorf, Hamburg, Germany; 2 Boston Scientific, Valencia, CA Spain; 3 Department of Neurology, Movement disorders & Neuromodulation section, Charité – University Medicine Berlin, Berlin, Germany

**Keywords:** Parkinson's disease, Parkinson's disease

## Abstract

Technological advances of Deep Brain Stimulation (DBS) within the subthalamic nucleus (STN) for Parkinson’s disease (PD) provide increased programming options with higher programming burden. Reducing the effort of DBS optimization requires novel programming strategies. The objective of this study was to evaluate the feasibility of a semi-automatic algorithm-guided-programming (AgP) approach to obtain beneficial stimulation settings for PD patients with directional DBS systems. The AgP evaluates iteratively the weighted combination of sensor and clinician assessed responses of multiple PD symptoms to suggested DBS settings until it converges to a final solution. Acute clinical effectiveness of AgP DBS settings and DBS settings that were found following a standard of care (SoC) procedure were compared in a randomized, crossover and double-blind fashion in 10 PD subjects from a single center. Compared to therapy absence, AgP and SoC DBS settings significantly improved (*p* = 0.002) total Unified Parkinson’s Disease Rating Scale III scores (median 69.8 interquartile range (IQR) 64.6|71.9% and 66.2 IQR 58.1|68.2%, respectively). Despite their similar clinical results, AgP and SoC DBS settings differed substantially. Per subject, AgP tested 37.0 IQR 34.0|37 settings before convergence, resulting in 1.7 IQR 1.6|2.0 h, which is comparable to previous reports. Although AgP long-term clinical results still need to be investigated, this approach constitutes an alternative for DBS programming and represents an important step for future closed-loop DBS optimization systems.

## Introduction

In advanced Parkinson’s disease (PD), deep brain stimulation (DBS) of the subthalamic nucleus (STN) effectively improves PD symptoms and quality of life^1,2^. Innovative technologies such as directional DBS leads and “multiple independent current control” (MICC) enable to shape precisely stimulation fields in a three-dimensional space by the fine control of current fractionalization on each of the lead’s electrodes. These innovations can be useful in patients with suboptimally placed DBS leads, as they allow to widen therapeutic windows and reduce side effects by redirecting stimulation fields towards therapeutic targets and away from structures that provoke side effects^3–6^. However, the multitude of possible stimulation settings available with directional DBS leads and MICC outnumbers the stimulation settings that can be feasibly explored with the classical monopolar review approach^7,8^, where exploration time burden is proportional to the number of clinically assessed stimulation settings.

To use the full potential of DBS and facilitate the programming of stimulation settings, techniques supplementary to clinical testing have been suggested. Besides image based^9–16^ and electrophysiological^17–20^ centered approaches, semi-automatic algorithms using external sensors as input are alternatives for the optimization of DBS settings^21–23^. Recently, an algorithm-guided-programming (AgP) designed for a MICC DBS system with ring and directional DBS leads has proven to reduce DBS optimization burden in PD patients^22,23^. In two exploratory studies, the DBS settings suggested by the algorithm achieved acute improvement of total Unified Parkinson’s Disease Rating Scale part III (UPDRS III) scores comparable to the improvement achieved by settings found following a standard of care (SoC) procedure^22,23^. However, SoC DBS settings resulted in a better subject´s global clinical state, which was apparent for the investigators but remained uncaptured by standardized items in the UPDRS III survey. The better performance of SoC over algorithm settings might be attributable to the use of a single bradykinesia feedback as algorithm’s input, which might be unable to represent the subject’s global clinical state. In the present study, we investigated an updated version of the previously reported algorithm that incorporates the use of multiple symptom scores as feedback input by introducing the concept of a total weighted score that aims to: (1) better capture the subject´s global clinical state and (2) compensate for the differentiated response of PD symptoms to DBS.

## Results

### Patient demographics

Between August 2019 and October 2020, 10 subjects (one female, nine males) were enrolled, comprising the 20 hemispheres analyzed in this study. The cohort consists of one left and nine right-handed subjects with an age of 54.5 IQR 54.0|59.0 years at the date of the study visit. Disease duration was 13.0 IQR 12.0|14.0 years with 11.0 IQR 7.0|11.0 years since the start of medication and 8.0 IQR 7.6|12.1 months since DBS surgery. Further patient characteristics are described in Supplementary Table 1.

### Algorithm inputs and burden

Based on the baseline scores assessed at the preliminary assessment phase of the study visit, 3.0 IQR 3.0|3.5 symptoms were selected per hemisphere to generate the total weighted score. From those symptoms, 2.0 IQR 2.0|2.5 were manually assessed by the clinician and 1.0 IQR 1.0|1.0 by the sensor. Upper limb rigidity was always scored, 2.0 IQR 1.0|2.0 symptoms were bradykinesia related and 0.0 IQR 0.0|1.0 symptoms were tremor related.

**Fig. 1:**
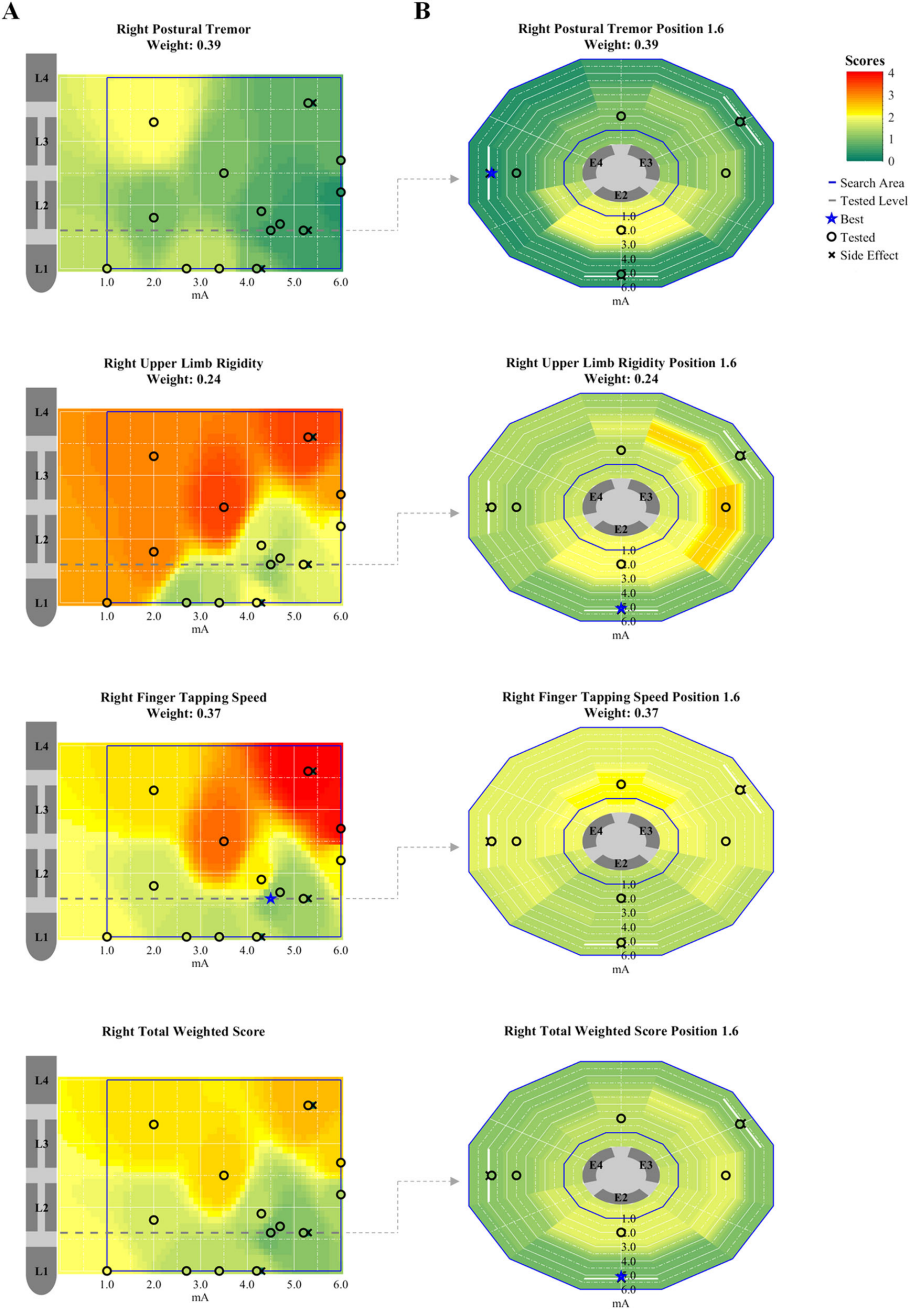
Exemplary score maps showing responses to AgP DBS settings for subject’s 0309-010 right body side symptoms (postural tremor, upper limb rigidity, finger tapping speed, and total weighted score). Score maps were generated for the **A** ring and **B** directional mode stages. Dashed lines on the ring mode stage score maps indicate the level at which the directional mode stage was performed. Red colored areas represent high symptom load and less DBS efficacy, whereas dark green colored areas low symptom load and high DBS efficacy. Note that score maps differed slightly between individual symptoms.

Clinical responses to the tested DBS settings were variable among the assessed symptoms (e.g., right body side of subject 0309-010; Fig. 1). For 19 hemispheres, AgP DBS settings leading to the best scores for the combined, total weighted score differed to the DBS settings leading to the best scores for the individual, single symptoms (e.g., Fig. 1 blue star markers and Supplementary Table 2 Setting Number 7, 9, 12, and 15). These differences comprised discrepancies in the suggested stimulation amplitude (1 hemisphere), in the electrode configuration (1 hemisphere) or in both parameters (17 hemispheres).

**Fig. 2:**
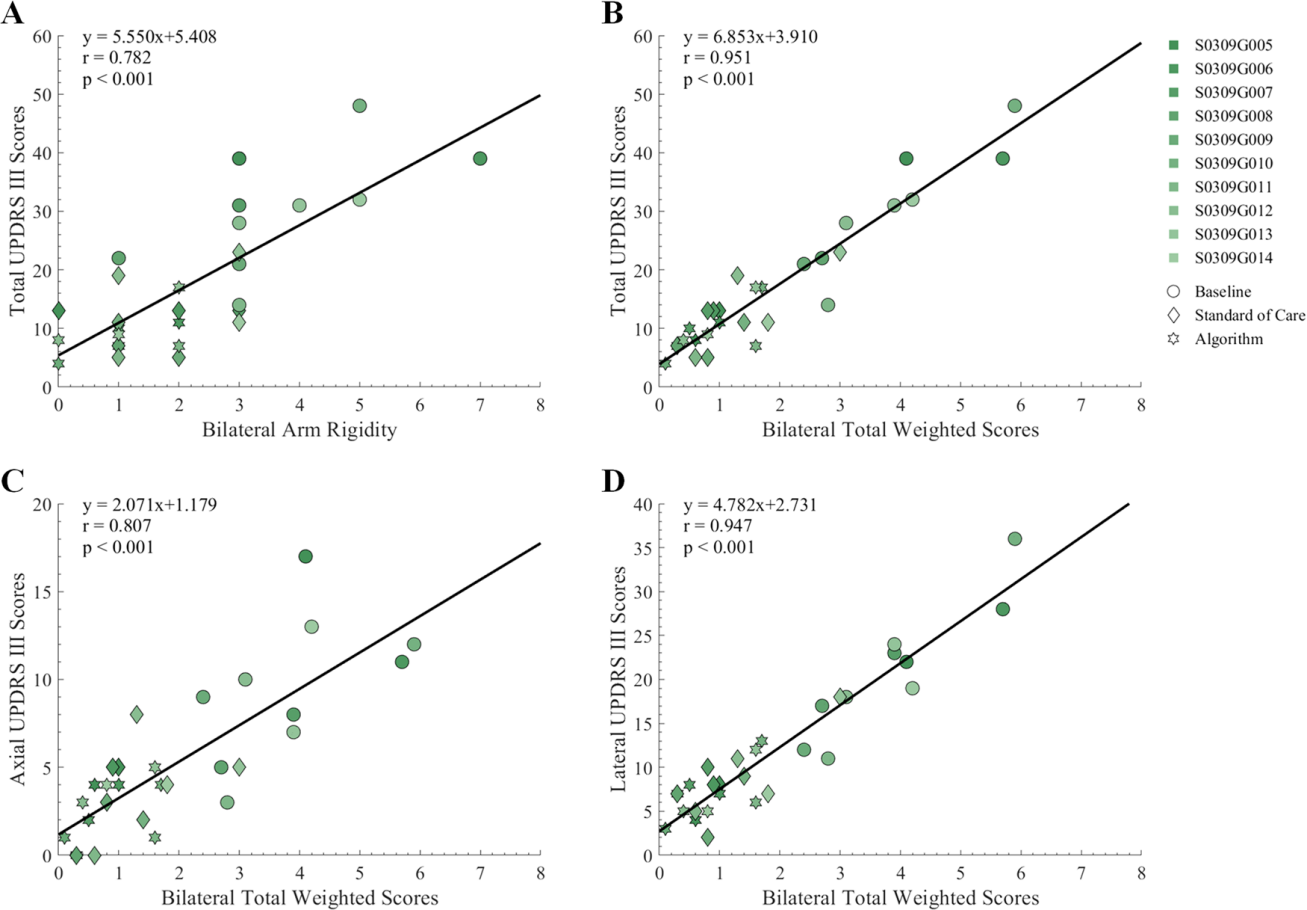
Correlation of bilateral upper limb rigidity scores and total weighted scores with UPDRS III scores of all subjects (*n* = 10). **A** Bilateral upper limb rigidity scores and **B** Bilateral total weighted scores correlation with total UPDRS III scores. **C** Axial symptoms UPDRS III scores and **D** Lateral symptoms UPDRS III scores correlation with bilateral total weighted scores.

Bilateral upper limb rigidity and total weighted scores were significantly (*p* ≤ 0.001) and strongly correlated with total UPDRS III scores (*r* = 0.782 and *r* = 0.951, respectively; Fig. 2A, B). Moreover, the correlations between bilateral total weighted scores and total scores of the axial and lateral items of the UPDRS III survey were also significant (*p* ≤ 0.001) and strong (*r* = 0.807 and *r* = 0.947, respectively; Fig. 2C, D).

In 13 of the 20 hemispheres, the AgP reached the directional mode exploration stage before it converged. For each subject, the AgP needed 1.7 IQR 1.6|2.0 h and 37.0 IQR 34.0|37 steps to converge. However, in 15 hemispheres the best stimulation settings were found before the algorithm converged (e.g., Supplementary Table 2), which led to a potential optimization burden of 1.4 IQR 1.0|1.7 h and 27.5 IQR 19.0|31.0 steps per subject (Supplementary Fig. 1).

### Therapy effectiveness

Compared to baseline (i.e., MED OFF/SoC DBS OFF), both SoC and AgP DBS settings improved the clinical condition of the subjects (Supplementary Fig. 1 and Supplementary Table 3). Total UPDRS III scores yielded by SoC and AgP DBS settings had a SoC-AgP difference of 2 IQR 0|5 points (*p* = 0.172) and their score similarity was 0.9 IQR 0.9|0.9. Improvement in total UPDRS III scores for SoC was 66.2 IQR 58.1|68.2% (*p* = 0.002) and 69.8 IQR 64.6|71.9% (*p* = 0.002) for AgP (Supplementary Fig. 1). These stimulation-induced improvements correspond respectively to 87.0 IQR 80.6|136.4% and 100.0 IQR 84.2|136.4% of the improvement in the preoperative Levodopa challenge. Compared to baseline, bilateral upper limb rigidity scores improved 53.4 IQR 25.0|71.4% (*p* = 0.008) for SoC and 69.1 IQR 50.0|100.0% (*p* = 0.004) for AgP (Supplementary Fig. 1). Sensor assessed bradykinesia scores improved 31.1 IQR 8.1|48.1% (*p* = 0.020) for SoC and 44.0 IQR 0.9|53.1% (*p* = 0.027) for AgP and sensor assessed rest tremor scores improved 98.8 IQR 61.9|100.0% (*p* = 0.078) for SoC and 60.5 IQR 12.1|92.3% for AgP (*p* = 0.059) (Supplementary Fig. 1).

### Stimulation settings

**Fig. 3:**
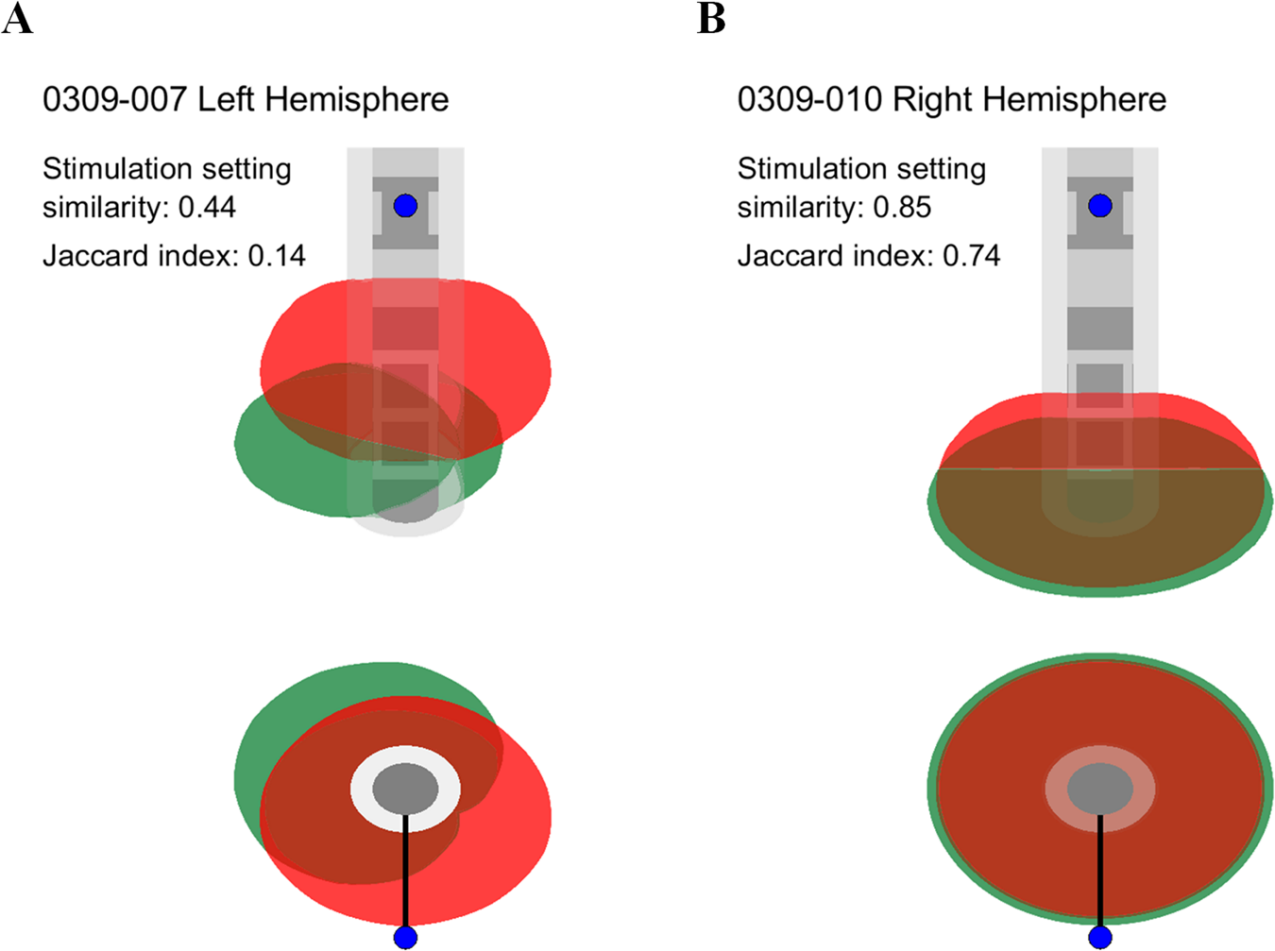
Exemplary VTAs generated from SoC and AgP DBS settings. Front and top view (upper and lower panels, respectively) of the DBS lead. Grey volume around the lead represents its encapsulation layer. **A** Subject’s 0309-007 left hemisphere SoC and AgP DBS settings have low similarity quantified by their low stimulation setting similarity and VTA Jaccard index (0.44 and 0.14, respectively). **B** Subject’s 0309-010 right hemisphere SoC and AgP DBS settings have high similarity quantified by their high stimulation setting similarity and VTA Jaccard index (0.85 and 0.74, respectively). SoC and AgP DBS settings for both subjects are included in Table 1.

The stimulation amplitude for the 20 analyzed hemispheres was 3.3 IQR 2.2|3.8 mA for SoC and 3.3 IQR 2.8|4.3 mA for AgP, which led to a SoC-AgP amplitude difference of −0.2 IQR −1.4|0.6 mA; *p* = 0.271 (Table 1). The electrode configuration was set to deliver ring mode stimulation in seven hemispheres for SoC and 11 hemispheres for AgP DBS settings. In four of the 13 hemispheres in which the AgP reached the directional mode exploration stage, directional mode DBS settings led to the best total weighted scores. In 12 hemispheres, the stimulation mode for SoC differed to that for AgP (e.g., subject 0309-005 left hemisphere; Table 1). The normalized similarity of stimulation settings between SoC and AgP was 0.5 IQR 0.4|0.6. The modeled Volume of Tissue Activated (VTA) generated for each of the DBS settings had a volume of 119.1 IQR 62.8|138.9 mm^3^ and 113.6 IQR 88.8|156.9 mm^3^ for SoC and AgP, respectively. The SoC-AgP VTA difference was −10.8 IQR −58.4|33.2 mm^3^; *p* = 0.296. The Jaccard index used to quantify the similarity between SoC and AgP VTAs was 0.3 IQR 0.2|0.4, indicating that for most of the SoC and AgP DBS settings there is little overlap between their VTAs (e.g., Fig. 3A).

**Table 1:** Comparison of study subjects’ standard of care (SoC) and algorithm-guided-programming (AgP) DBS settings.

Subject ID	Hemisphere	SoC DBS settings				AgP DBS settings			
		Amplitude (mA)	Frequency (Hz)	Electrode configuration	Stimulation mode	Amplitude (mA)	Frequency (Hz)	Electrode configuration	Stimulation mode
0309-005	Left	3.5	159	E2: −100%	Directional	2.8	130	E1: −100%	Ring
	Right	2.5	159	E5: −100%	Directional	2.9	130	E2: −8%, E4: −22%, E5: −18%, E7: −52%	Directional
0309-006	Left	3.7	159	E2: −100%	Directional	3.5	130	E1: −100%	Ring
	Right	2.0	159	E7: −100%	Directional	4.3	130	E5: −8%, E6, E7: −6%, E8: −80%	Ring
0309-007	Left	2.1	130	E5: −100%	Directional	2.7	130	E5: −60%, E8: −40%	Directional
	Right	2.2	130	E3: −100%	Directional	3.5	130	E1: −50%, E2: −12%, E4: −38%	Directional
0309-008	Left	2.7	185	E2: −34%, E3, E4: −33%	Ring	5.0	130	E2: −2%, E4: −8%, E5: −22%, E7: −68%	Directional
	Right	1.0	185	E2: −34%, E3, E4: −33%	Ring	3.0	130	E1: −100%	Ring
0309-009	Left	3.9	130	E4: −100%	Directional	2.8	130	E1: −100%	Ring
	Right	1.7	130	E3: −100%	Directional	3.1	130	E1: −100%	Ring
0309-010	Left	4.6	130	E1: −70%, E2, E3, E4: −10%	Ring	5.1	130	E1: −40%, E2: −60%	Directional
	Right	3.8	130	E1: −100%	Ring	3.7	130	E1: −70%, E2, E3, E4: −10%	Ring
0309-011	Left	5.0	130	E5: −100%	Directional	4.2	130	E2: −75%, E3: −25%	Directional
	Right	4.0	130	E4: −100%	Directional	3.6	130	E1: −20%, E2: −28%, E3, E4: −26%	Ring
0309-012	Left	2.3	130	E5: −34%, E6, E7: −33%	Ring	2.0	130	E1: −30%, E2: −70%	Directional
	Right	2.1	130	E5: −34%, E6, E7: −33%	Ring	2.0	130	E2: −70%, E5: −30%	Directional
0309-013	Left	3.6	130	E6: −100%	Directional	4.5	130	E1: −40%, E2, E3, E4: −20%	Ring
	Right	3.7	130	E3: −100%	Directional	5.4	130	E1: −100%	Ring
0309-014	Left	4.0	130	E2: −34%, E3, E4: −33%	Ring	2.5	130	E1: −70%, E2, E3, E4: −10%	Ring
	Right	3.1	130	E2: −100%	Directional	2.0	130	E1: −20%, E2: −80%	Directional

For both SoC and AgP DBS settings, pulse width was set to 60 µs for all hemispheres. Note that AgP’s first stage explored the vertical plane by testing ring mode DBS settings along the lead’s axis and continued with a second stage exploring the horizontal plane at the level of the best ring mode DBS setting by testing directional mode DBS settings around the lead’s axis.

**Fig. 4:**
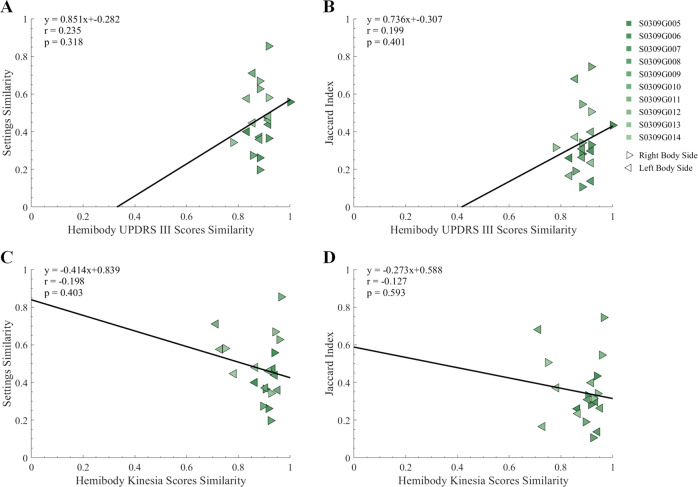
Non-significant correlation of similarities between SoC and AgP clinical effects and their DBS settings. Correlation of **A** settings similarity and **B** VTA Jaccard index with hemibody UPDRS III scores similarity (*n* = 20). Correlation of **C** settings similarity and **D** VTA Jaccard index with hemibody Kinesia™ One scores similarity (*n* = 20).

The similarity between SoC and AgP UPDRS III scores was not significantly correlated with neither their similarity in stimulation settings (*r* = 0.235, *p* = 0.318; Fig. 4A) nor with their VTA Jaccard index (*r* = 0.199, *p* = 0.401; Fig. 4B). This lack of significant correlation was also observed between the similarity of Kinesia™ One scores and the similarity in stimulation settings (*r* = −0.198, *p* = 0.403; Fig. 4C) and the VTA Jaccard index (*r* = −0.127, *p* = 0.593; Fig. 4D). Further results are presented in Supplementary Data 1 and Supplementary Figs. 2 and 3.

## Discussion

CLOVER-DBS is a novel semi-automatic, closed-loop iterative computer-algorithm facilitating the initial programming of DBS settings for PD patients. This version of the algorithm supports the programming of DBS settings for directional leads and incorporates the use of multiple symptoms by introducing the concept of a total weighted score, which is used as the algorithm’s feedback input and aims to better capture the subject’s global clinical state. Algorithm-suggested DBS settings result in significant acute reduction of PD motor symptoms, with slightly better immediate results compared to SoC DBS settings and with a programming time comparable to the duration of a typical initial DBS programming session^24^. Symptoms selected to calculate the total weighted score show differentiated DBS responses that lead to different potential therapeutic DBS settings.

The score maps generated by the algorithm show that there are defined regions within the stimulation space that had the potential to lead to therapy effective DBS settings. Although these regions differ among the assessed symptoms, a certain overlap is often observed. This observation is aligned with previous works that have generated probabilistic stimulation maps (i.e., sweet spots) based on the aggregation of several VTAs for movement disorders such as Parkinson’s disease and Dystonia^12,25^. It has been suggested that the therapeutic response of multiple symptoms to DBS settings should be assessed to better characterize the patient’s global clinical state^23,26^. Rest tremor of the upper extremities, finger tapping and gait have been found to have strong correlations with total UPDRS III scores^27^. The total weighted score introduced in this work aims to capture this global clinical state by the weighted combination of several relevant symptoms that account for a fraction of the total UPDRS III scores. Importantly, the generation of the total weighted score is agnostic to the type of assessed responses given that they have a defined value range. This feature of the total weighted score allows to combine manually assessed and sensor derived signals. Compared to bilateral upper limb rigidity scores, total weighted scores show a stronger correlation with total UPDRS III scores and a strong correlation with axial symptoms, for which the optimization of DBS settings might be more challenging.

The algorithm constitutes a naïve new paradigm to optimize DBS settings and because of this, it often suggests stimulation amplitudes or electrode configurations that might not be intuitive for clinicians but that are nevertheless necessary to define the available stimulation space that could yield therapy effective DBS settings. The DBS optimization was often attained before the AgP finally converged. In clinical practice, it would be possible to prematurely stop the AgP when clinician and patient are satisfied with the AgP-derived DBS settings. Although previous studies have contributed with evidence about symptom specific stimulation sweet spots in the STN^13,25^, the algorithm in this work aims to find a “one-fits-all” DBS setting that is effective for all symptoms used to calculate the total weighted score despite the possibility that this solution might not be the optimal for each of these symptoms. Based on the metrics proposed in this work, AgP DBS settings have a low similarity with SoC DBS settings despite their similar clinical results. This small similarity probably originates from the differences in the SoC and AgP electrode configurations rather than from their stimulation amplitudes, as in contrast to SoC, AgP DBS settings often involve the activation of multiple electrodes and two electrode levels and neither the stimulation amplitude nor the volume of the generated VTAs from both DBS settings had significant differences.

The study has certain limitations as a small sample size of ten subjects and 20 hemispheres from a single center with one-day study visit precluding long-term observations. Nonetheless, these sample sizes are comparable to other DBS exploratory studies and previous similar works^21–23^. There is also a selection bias, as only less severely affected subjects tolerating withdrawal of medication for several hours participated in the study. The SoC DBS settings of the subjects yielded high beneficial clinical effects, thus there might be a ceiling effect masking the superiority of AgP over SoC. Future studies should consider longer term observation of AgP clinical effects and subjects with suboptimal clinical results due to DBS.

The AgP approach investigated here constitutes a feasible alternative for the semi-automatic optimization of DBS settings and could be used to guide future closed-loop DBS optimization systems. Next versions of the algorithm should incorporate electrophysiological and anatomical information to constrain the stimulation space to be explored aiming to further reduce the programming burden.

## Methods

### Study design

CLOVER-DBS (**C**losed **Lo**op Programming E**v**aluation Using **E**xternal **R**esponses) is an exploratory, prospective, multicenter study with a randomized, crossover and double-blind design^23^ (ClinicalTrials.gov Identifier NCT03037398). Acute therapeutic effects of SoC DBS settings on motor performance are compared with the effects of DBS settings obtained following an AgP approach. Three exploratory endpoints of the study quantify the difference in therapy effectiveness by comparing (1) total UPDRS III scores, (2) bilateral upper limb rigidity scores obtained as part of the UPDRS III and (3) bradykinesia and tremor scores measured by an external finger mounted sensor (Kinesia™ One, Great Lakes Neurotech, Cleveland, OH) with a proprietary scoring algorithm that assigns scores from 0 to 4 to each assessment^28,29^. The Kinesia™ One system assesses the speed, amplitude and rhythm of finger tapping and hand grasping tasks for bradykinesia as well as rest tremor of the upper limbs during a time period of 15 s. Additionally, time burden and number of stimulation settings (i.e., steps) needed for the AgP to converge were recorded.

### Participants

The analyzed cohort originated from a single center (University Medical Center Hamburg-Eppendorf, Hamburg, Germany, UKE). The study was approved by the local ethics committee in Hamburg, Germany (PV MC-089/18) and conducted in agreement with the Code of Ethics of the World Medical Association (Declaration of Helsinki, 2018). Written informed consent to publish was obtained. Inclusion criteria were (1) diagnosis of bilateral idiopathic PD with the presence of bradykinesia and/or tremor, (2) on-label implantation of the Vercise DBS directional system (Boston Scientific, Valencia, California, USA) in the STN, (3) unchanged SoC DBS settings for at least 4 weeks, (4) total UPDRS III scores ≥25 points in the preoperative medication off (MED OFF) state, and (5) improvement of PD symptoms as a result of DBS in the MED OFF state defined as ≥25% reduction in total UPDRS III scores. Exclusion criteria were (1) candidates with major psychiatric comorbidities including unrelated clinically significant depression and (2) usage of multiple frequencies and/or activation of non-adjacent electrodes for the SoC DBS settings.

### Study workflow

**Fig. 5:**
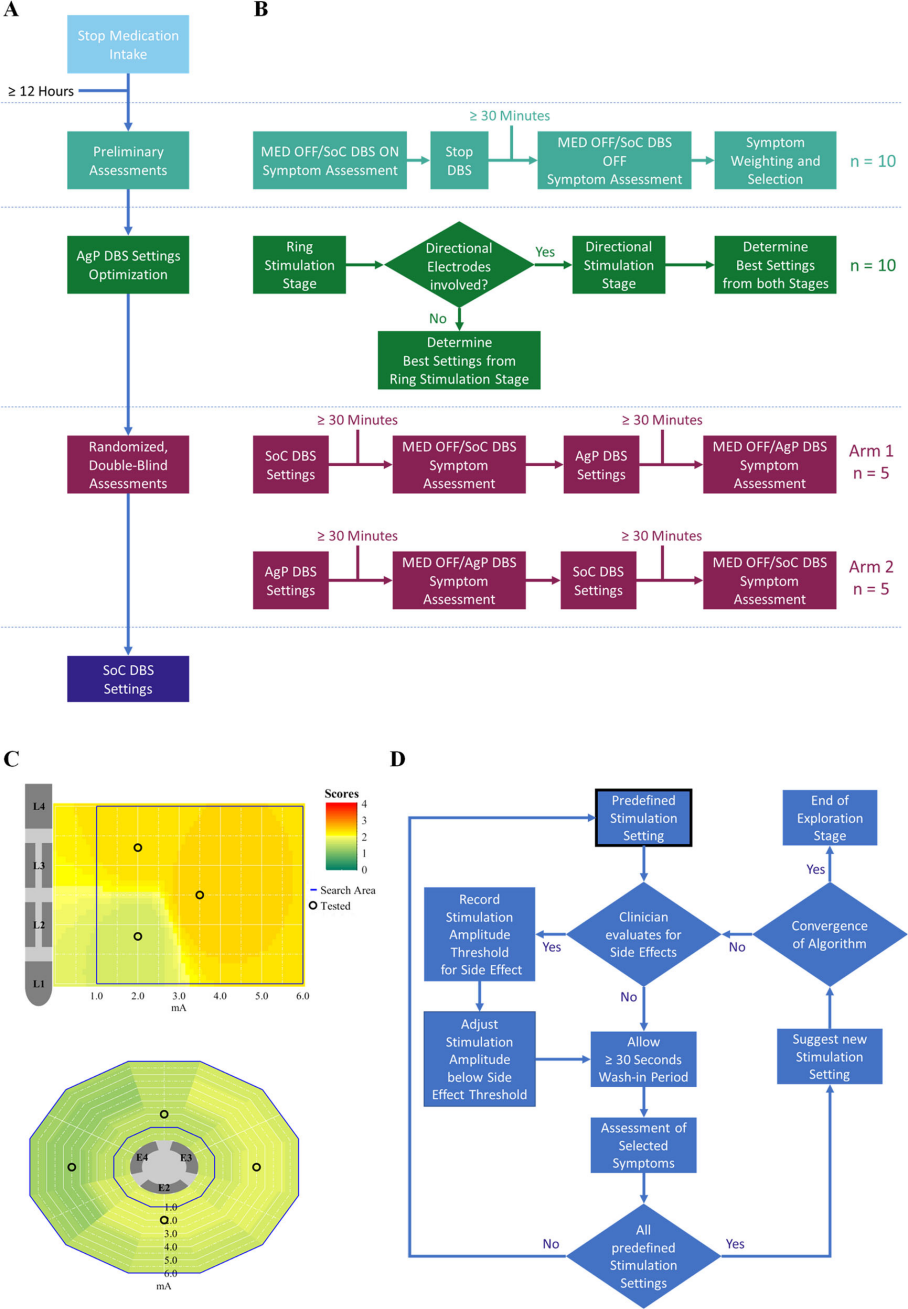
Overview of main phases and specific task of the study. Block diagrams of **A** the main phases of the study visit and **B** the specific tasks performed at each of them. **C** Examples of score maps for the predefined exploration points at the two AgP exploration stages. The algorithm starts with the same standard predefined stimulation settings, which were three and four for the ring and directional mode exploration stages, respectively (circles on panels). **D** Block diagram for both AgP exploration stages. Ring mode stimulation constitutes the first AgP exploration stage. If the ring mode stimulation setting leading to the best clinical effect involves the activation of directional electrodes, the algorithm continues with the second exploration stage, which involves directional mode stimulation settings. The black contoured block indicates the start of the corresponding exploration stage.

CLOVER-DBS consists of one study visit with three main phases: (1) preliminary assessments, (2) AgP DBS settings optimization, and (3) randomized, double-blind assessments (Fig. 5A, B). The study was executed by two investigators, one clinician assessed the motor symptoms while the other investigator operated the algorithm. Subjects arrived at the study visit in the MED OFF condition after withdrawal of antiparkinsonian medication for at least 12 h, in which preliminary clinical assessments were performed. AgP was performed individually and in arbitrary order for each brain hemisphere, while DBS for the opposite hemisphere was turned off. Pulse width and frequency were set to 60 µs and 130 Hz. The clinical effect of DBS settings suggested by the algorithm was assessed on the contralateral bodyside after a wash-in period of 30 s. Once the algorithm completed the optimization of DBS settings for both hemispheres, the performance of AgP and SoC DBS settings was compared in random order and in a double-blind fashion based on the assessed severity of PD symptoms after a DBS wash-in period of 30 min. Random allocation sequence was implemented by sequentially numbered containers generated by Boston Scientific in advance.

While the burden associated to finding SoC DBS settings is not reported because it could not be reliably estimated based on the patient’s clinical files, the AgP “convergence burden” is defined as the time and number of evaluation steps of different stimulation settings suggested by the algorithm during the AgP DBS optimization phase until AgP converges. Moreover, a potential “optimization burden” was defined as the time and number of evaluation steps needed by the algorithm to suggest the clinically best DBS settings (based on the Total Weighted Score) for both hemispheres (Supplementary Table 2).

### Symptom selection and weighting

Based on the scores obtained in the preliminary assessments phase, weights between 0 and 1 with a 0.001 resolution were assigned to lateralized symptoms using a proprietary method that considers their severity in the absence of therapy and their potential improvement due to therapy (i.e., MED OFF/SoC DBS OFF and MED OFF/SoC DBS ON conditions, respectively). For instance, a symptom improving from a score of 4 to 0 will have a weight of 1.0, whereas a symptom improving from 2 to 0 will have a weight of 0.50 and a symptom improving from 2 to 1 will have a weight of 0.125. The symptoms were ranked by their weight and a maximum of four symptoms were selected per hemisphere. Symptom scores (point resolution of 0.1) and their normalized weights were used to generate a total weighted symptom score, which was used as input for the algorithm.

### Computer algorithm

The previously described computer algorithm^23^ is a stand-alone graphic user interface created in MATLAB R2020a (Mathworks, Natick, Massachusetts, USA). The algorithm has been developed to be compatible with the Boston Scientific Cartesia directional lead. The algorithm consists of two iterative and sequential exploration stages, in which DBS settings are tested within the corresponding two-dimensional stimulation space (Fig. 5C). The first exploration stage of the algorithm is the “Ring mode” stimulation, where several DBS settings at different vertical positions along the axis of the DBS lead are tested. If the vertical position (i.e., ring mode DBS setting) leading to the best clinical effect involve the activation of directional electrodes, the second exploration stage of the algorithm involves directional mode stimulation at this vertical position after algorithm convergence at the first stage. In the directional mode stage, several DBS settings at different rotation angles around the DBS lead are tested (Fig. 5C, D). Due to the naïve nature of the algorithm, at the beginning of each exploration stage it explores predefined standard points (i.e., DBS settings) distributed over the corresponding two-dimensional stimulation space. These predefined points were the same for all subjects, comprising three points for the ring mode stage and four for the directional mode stage. Once the predefined points are explored, the algorithm suggests new stimulation settings based on the assessed clinical responses to the previous settings. At each step (i.e., iteration), the DBS settings corresponding to the explored point were programmed on the subject’s IPG allowing a wash-in period of ≥30 s before the selected symptoms’ scores were recorded and used to generate the total weighted score, which served as input for the algorithm.

Based on all recorded scores and side effects, two-dimensional score maps were generated for each of the selected symptoms and the total weighted score (Figs. 1 and 5C). These score maps contain the potential clinical effect at each point on the corresponding stimulation space, as well as stimulation amplitude boundaries for regions around the vertical position or rotation angles where side effects were observed, which further constrained the two-dimensional stimulation space to be explored.

Based on the potential clinical effects for the total weighted score and the distance between all previously explored points, the algorithm determined the next point to explore by means of a proprietary weighting method. This weighting method allows the exploration of regions that are far from each other at the beginning, and then focus on regions with the best potential of therapeutically effective DBS settings. Once the distance between the new suggested and the explored points is below a predefined threshold, the algorithm converges (Fig. 5D).

### Similarity of stimulation settings and scores

The similarity of SoC and AgP DBS settings was quantified for each hemisphere by two methods. For both methods, similarity values ranged from 0 to 1, with values of 1 indicating identical stimulation settings and values of 0 completely different stimulation settings. The first method quantified the SoC|AgP settings similarity based on the ratio of their stimulation amplitudes and the normalized Euclidean distance of their electrode configurations (Eqs. 1–3). The second method quantified the similarity of the resulting VTAs from both stimulation settings by calculating their Jaccard index (Eq. 4). A description of the method used to generate the VTAs has been reported previously^30^.

1 $$SetSim = AmpRat^ \ast \left( {1 - NormElcConfDist} \right)$$

Where:

SetSim: SoC|AgP stimulation settings similarity.

AmpRat: SoC|AgP stimulation amplitude ratio.

NormElcConfDist: normalized SoC|AgP electrode configuration Euclidean distance.

2 $$AmpRat = \frac{{min\left( {StimAmp_{SoC},StimAmp_{AgP}} \right)}}{{max\left( {StimAmp_{SoC},StimAmp_{AgP}} \right)}}$$

Where:

AmpRat: SoC|AgP Stimulation amplitude ratio.

StimAmp_SoC_: SoC stimulation amplitude.

StimAmp_AgP_: AgP stimulation amplitude.

3 $$NormElcConfDist = \frac{{\root {2} \of {{\mathop {\sum }\nolimits_{i = 1}^N \left( {SoC\;ElecAct_i - AgP\;ElecAct_i} \right)^2}}}}{{\sqrt 8 }}$$

Where:

NormElcConfDist: Normalized SoC|AgP electrode configuration Euclidean distance.

*N*: total number of electrodes on the lead, including the IPG case.

SoC ElecAct_i_: Normalized SoC activation of electrode i.

AgP ElecAct_i_: Normalized AgP activation of electrode i.

4 $$JacIdx = \frac{{VTA_{SoC} \cap VTA_{AgP}}}{{VTA_{SoC} \cup VTA_{AgP}}}$$

Where:

JacIdx: SoC|AgP VTAs Jaccard Index.

VTA_SoC_: SoC volume of tissue activated.

VTA_AgP_: AgP volume of tissue activated.

For each body side, the similarity of scores yielded by SoC and AgP DBS settings was quantified for UPDRS III as well as Kinesia™ One scores. The quantification was based on the SoC|AgP score Euclidean distance for each of the assessed items (i.e., symptoms) of the corresponding test (Eq. 5). This similarity metric had a value range from 0 to 1, with values of 1 indicating identical scores for all symptoms and values of 0 the maximum score difference (i.e., 4) for all symptoms.

5 $$ScrSim = 1 - \frac{{\root {2} \of {{\mathop {\sum }\nolimits_{i = 1}^N \left( {SoC\;Scr_i - AgP\;Scr_i} \right)^2}}}}{{\sqrt {16 \ast N} }}$$

Where:

ScrSim: normalized SoC|AgP score similarity.

*N*: total number of symptoms assessed.

SoC Scr_i_: score for symptom i yielded by SoC DBS settings.

AgP Scr_i_: score for symptom i yielded by AgP DBS settings.

### Statistics

CLOVER is an exploratory study and due to the small sample size, results are reported as median and 25% | 75% interquartile range (IQR). Wilcoxon signed rank test was used to test for difference significance when comparing conditions (*p* < 0.05). Pearson’s correlation was used to quantify the relationship between variables and its strength was categorized as strong 1.0 ≥ |*r*| ≥ 0.7, moderate 0.7 > |r| ≥ 0.5 or weak 0.5 > |r| ≥ 0.3. All analyses were performed on MATLAB R2020a.

## Supplementary information

Supplementary Files

## Data Availability

The data that support the findings of this study are available from Boston Scientific but restrictions apply to the availability of these data conditioned to Boston Scientific Data Sharing Policy and so are not publicly available. Data are however available from the corresponding author upon reasonable request and with permission of Boston Scientific. The data and study protocol for this clinical trial may be made available to other researchers in accordance with the Boston Scientific Data Sharing Policy (http://www.bostonscientific.com/en-US/data-sharing-requests.html).

## Code availability

The data analysis code from this clinical trial may be made available to other researchers in accordance with the Boston Scientific Data Sharing Policy (http://www.bostonscientific.com/en-US/data-sharing-requests.html).
