# Supplementary material for: Multiple input algorithm-guided Deep Brain stimulation-programming for Parkinson’s disease patients
Source: NPJ Parkinsons Dis. 2022 Oct 29;8:144. doi: 10.1038/s41531-022-00396-7 (PMC9617933; doi:10.1038/s41531-022-00396-7)
Supplement: Supplementary file 1 — Supplementary Files [file 41531_2022_396_MOESM1_ESM.pdf]

Supplementary Data 1

For the seven hemispheres in which SoC DBS settings delivered ring mode stimulation, SoC settings were projected onto the score maps generated in the AgP phase. The resulting coordinates of this projection were used to estimate the DBS response of the total weighted scores and all the symptoms used to generate it at the randomized, double-blind assessment phase of the study. The assessed-estimated score difference for SoC DBS settings was -0.3 IQR -0.9 | 0.1 points; $p = 0.022$ and their correlation was week ($r = 0.472$, $p = 0.010$; Supplementary Figure 2A). In the case of AgP DBS settings, the difference in the scores assessed at the AgP phase and the randomized, double-blind assessments phase was of 0.0 IQR -0.2 | 0.3 points; $p = 0.863$, and their correlation was weak ($r = 0.498$, $p \leq 0.001$; Supplementary Figure 2B). For baseline as well as for SoC and AgP DBS settings assessments, UPDRS III scores showed significant ($p \leq 0.001$) and strong correlations with sensor assessed scores for total and hemibody (i.e., lateralized symptoms from each body side) scores ($r = 0.858$ and $r = 0.773$, respectively; Supplementary Figure 3).

Supplementary Figure 1

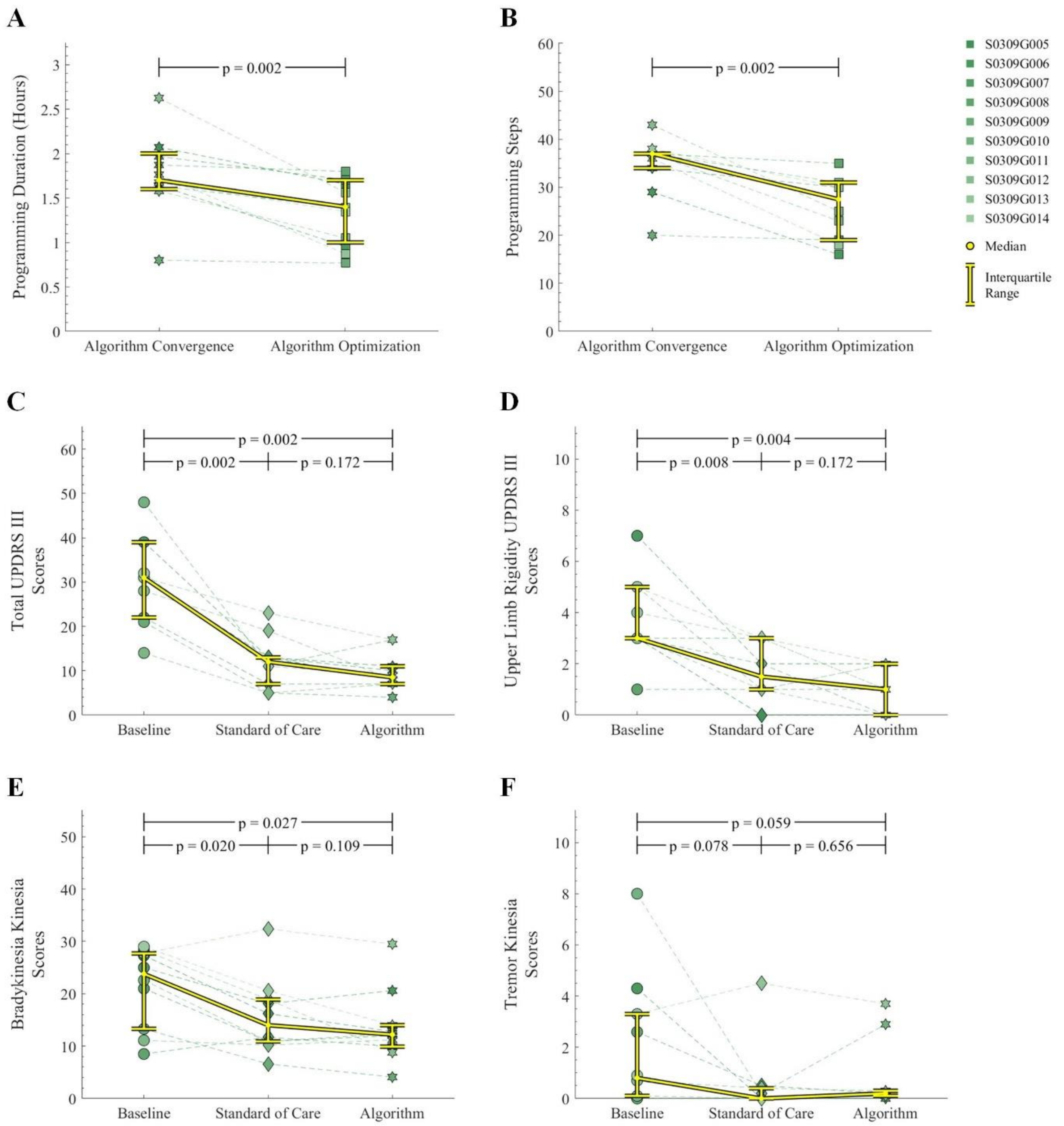

Programming burden for AgP and therapy effectiveness of SoC and AgP DBS settings based on manual and sensor assessed scores. (A) Duration of AgP exploration of DBS settings until algorithm convergence (stop of AgP exploration once the distance between the new suggested and previous explored DBS settings is below a predefined threshold) and algorithm optimization (optimal symptom control with DBS settings based on Total Weighted Scores). (B) Number of steps required for algorithm convergence and algorithm optimization. Effect of SoC and AgP DBS settings on (C) total UPDRS III scores and (D) manually assessed bilateral upper limb rigidity scores as part of the UPDRS III survey, as well as (E) sensor based bilateral bradykinesia scores comprising the assessment of speed, amplitude and rhythm of finger tap and hand grasping tasks and (F) sensor based bilateral rest tremor scores from the upper limbs.

Supplementary Figure 2

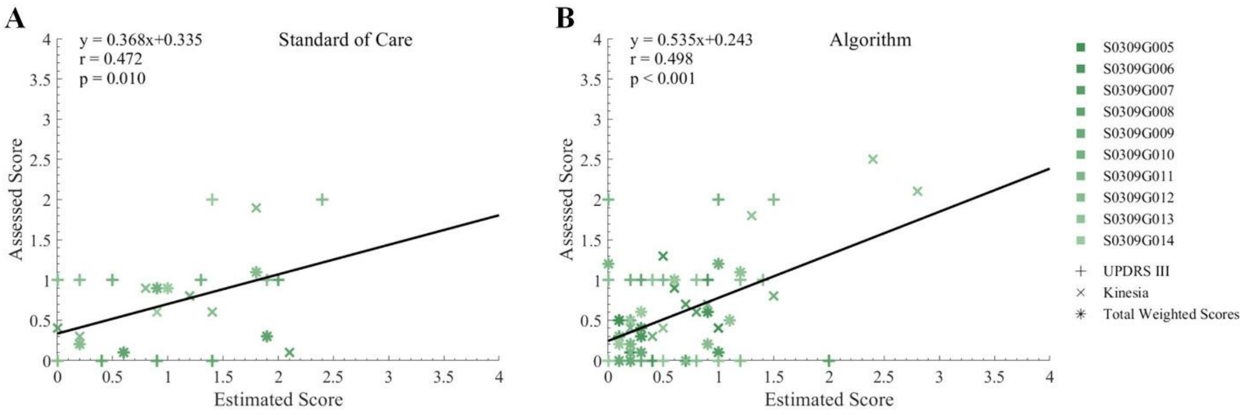

Correlation of scores estimated by the score maps and scores assessed at the randomized, double blind assessments phase of the study visit. (A) SoC DBS settings were projected on the corresponding two-dimensional score map. Based on the location of the SoC DBS settings on the map, scores for each of the assessed symptoms during the DBS optimization phase were estimated. (B) For AgP DBS settings, symptom scores on the score map correspond to the assessments performed during the DBS optimization phase.

Supplementary Figure 3

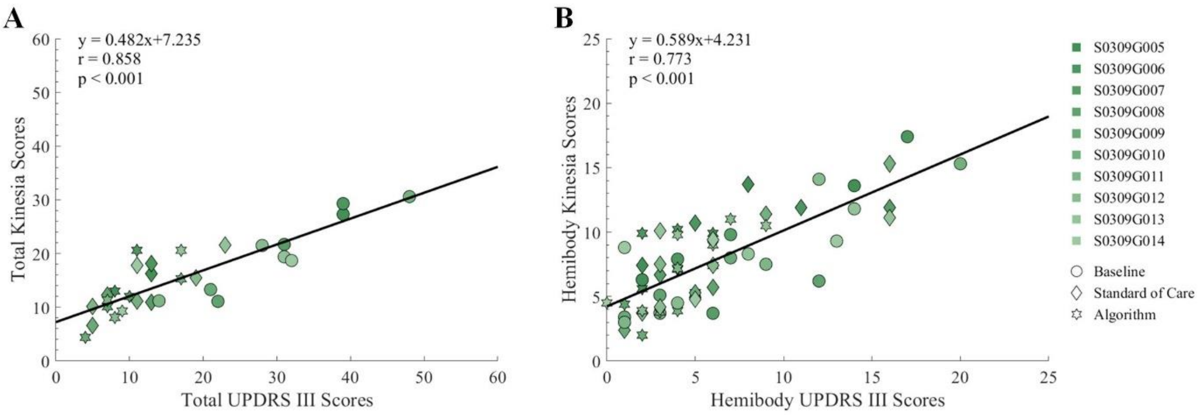

Correlation of assessed UPDRS III and Kinesia™ One scores. (A) Total UPDRS III and Kinesia™ One scores from each subject (n = 10). (B) Hemibody UPDRS III and Kinesia™ One scores (n = 20).

Supplementary Table 1

Subject ID	Gender	Age	Handedness	Years with Disease	Years with Medication	Months with DBS	Weeks since last DBS Settings Change	Preoperative Total UPDRS III	
								MED OFF	MED ON
0309-005	Male	56	Right	12	11	23	47	38	8
0309-006	Male	71	Right	13	11	12	23	39	21
0309-007	Male	59	Right	9	9	7	5	40	7
0309-008	Male	52	Right	13	5	7	11	24	14
0309-009	Female	55	Right	8	6	8	7	21	8
0309-010	Male	54	Right	14	11	12	6	43	8
0309-011	Male	54	Right	19	7	8	4	33	16
0309-012	Male	54	Left	24	15	6	4	22	5
0309-013	Male	69	Right	12	11	8	4	36	15
0309-014	Male	51	Right	14	11	7	4	25	9

Demographics of the study subjects. Preoperative Levodopa challenge was performed both in the off-medication state after overnight withdrawal of medication (MED OFF) and in the on-medication state after medication intake with supramaximal dose of 1.5x morning dosage (MED ON).

Supplementary Table 2

Setting Number	Amplitude (mA)	Vertical Position	Rotation (°)	Stimulation Mode	Upper Limb Rigidity	Rest Tremor	Leg Agility	Finger Tap Speed	Total Weighted Score	Side Effect	Time Stamp
1	2.0	1.8	N/A	Ring	1.0	1.0	1.0	0.8	1.0	No	09:34
2	2.0	3.3	N/A	Ring	1.2	2.0	1.3	1.1	1.5	No	09:36
--	3.1	2.5	N/A	Ring	--	--	--	--	--	Yes	09:39
3	3.0	2.5	N/A	Ring	0.8	0.8	0.7	0.6	0.8	No	09:44
--	4.0	1.5	N/A	Ring	--	--	--	--	--	Yes	09:46
4	3.5	1.5	N/A	Ring	1.4	1.3	0.8	0.9	1.1	No	09:49
--	3.5	4.0	N/A	Ring	--	--	--	--	--	Yes	09:51
5	3.0	4.0	N/A	Ring	0.8	0.9	0.5	0.8	0.7	No	09:54
--	2.9	3.4	N/A	Ring	--	--	--	--	--	Yes	09:57
6	2.8	3.4	N/A	Ring	0.9	0.4	0.5	0.9	0.6	No	10:00
7	2.8	3.1	N/A	Ring	0.8	0.9	0.3	0.7	0.7	No	10:04
8	2.0	3.4	0	Directional	1.4	0.4	0.9	0.9	0.9	No	10:08
--	2.1	3.4	90	Directional	--	--	--	--	--	Yes	10:10
9	2.0	3.4	90	Directional	1.0	1.0	0.8	0.6	0.9	No	10:13
10	2.0	3.4	180	Directional	0.9	0.7	1.0	0.8	0.9	No	10:16
--	2.9	3.4	270	Directional	--	--	--	--	--	Yes	10:18
11	2.8	3.4	270	Directional	1.2	0.8	1.0	1.0	1.0	No	10:21
--	2.8	3.4	0	Directional	--	--	--	--	--	Yes	10:23
12	2.7	3.4	0	Directional	0.9	0.2	0.8	0.7	0.6	No	10:25
--	3.6	3.4	180	Directional	--	--	--	--	--	Yes	10:27
--	3.5	3.4	180	Directional	--	--	--	--	--	Yes	10:29
13	3.2	3.4	180	Directional	1.1	1.3	0.7	0.9	1.1	No	10:31
14	1.0	3.4	240	Directional	1.0	0.8	1.4	0.7	1.0	No	10:35
15	1.0	3.4	120	Directional	1.2	0.2	1.5	1.2	1.0	No	10:38
16	2.7	3.4	0	Directional	2.0	0.3	0.7	0.8	1.0	No	10:41

Exemplary AgP DBS setting optimization for the left-brain hemisphere of subject 0309-007. DBS settings consist of a stimulation amplitude, vertical position and, in the case of directional mode DBS settings, a rotation angle. In this session, 16 different DBS settings ("Setting Number" column), comprising one hour and seven minutes (difference between first and last row on "Time Stamp" column), were necessary for algorithm convergence. However, the best Total Weighted Score, which defined algorithm optimization, was found for the 12th tested setting after 51 minutes from the start of the session. Best scores for Upper Limb Rigidity, Leg Agility were found for the 7th tested setting, whereas the best Finger Tap Speed and Rest Tremor scores were found for the 9th and 15th tested settings, respectively (thick frames). In case of score ties, the algorithm prefers stimulation settings with lower stimulation amplitude. DBS Settings leading to side effects are indicated by dashes on the Setting Number column. Vertical Position and Rotation indicates the location of the stimulation field along and around the lead's longitudinal axis, respectively.

Supplementary Table 3

Subject ID	Total UPDRS III			Upper Limb Rigidity			Kinesia™ One Bradykinesia			Kinesia™ One Rest Tremor		
	Baseline	SoC	AgP	Baseline	SoC	AgP	Baseline	SoC	AgP	Baseline	SoC	AgP
0309-005	39	13	8	3	0	0	27.3	16.2	12.8	0.0	0.0	0.2
0309-006	39	13	11	7	2	2	25.0	18.1	20.6	4.3	0.0	0.0
0309-007	31	13	10	3	3	1	21.0	10.9	12.0	0.7	0.0	0.0
0309-008	22	7	7	1	1	1	8.5	11.7	9.9	2.6	0.5	0.2
0309-009	21	5	4	3	2	0	13.3	6.6	4.1	0.0	0.0	0.3
0309-010	48	11	17	5	1	2	22.6	10.9	12.4	8.0	0.2	2.9
0309-011	14	5	7	3	1	2	11.1	10.2	11.2	0.1	0.0	0.1
0309-012	28	19	8	3	1	0	28.3	18.9	8.8	0.7	0.4	0.3
0309-013	31	23	17	4	3	2	27.7	32.4	29.5	3.3	4.5	3.7
0309-014	32	11	9	5	3	1	29.0	20.6	14.0	0.9	0.0	0.1
**Median**	**31**	**12**	**8.5**	**3**	**1.5**	**1**	**23.8**	**14.0**	**12.2**	**0.8**	**0.0**	**0.2**
**Interquartile Range**	**22 | 39**	**7 | 13**	**7 | 11**	**3 | 5**	**1 | 3**	**0 | 2**	**13.3 | 27.7**	**10.9 | 18.9**	**9.9 | 14.0**	**0.1 | 3.3**	**0.0 | 0.4**	**0.1 | 0.3**

Scores for the clinical effectiveness exploratory endpoints of the study. Upper Limb Rigidity (UPDRS III subitem) and Kinesia™ One scores are bilateral. Kinesia™ One Bradykinesia corresponds to the sum of finger tap and hand grasps speed, amplitude and rhythm, whereas Rest Tremor corresponds to upper limbs tremor amplitude.
